# Supplementary material for: Japanese Diet Indices and Nutrient Density in US Adults: A Cross-Sectional Analysis with NHANES Data
Source: Nutrients. 2024 Jul 26;16(15):2431. doi: 10.3390/nu16152431 (PMC11314486; doi:10.3390/nu16152431)
Supplement: Supplementary file 1 [file nutrients-16-02431-s001.zip › nutrients-3094647-supplementary.pdf]

SUPPLEMENTARY MATERIALS

As the corresponding food item of the Japanese diet indices, the selected food codes and a description of the food and The What We Eat in America (WWEIA) food category are shown in **Table S1** for “rice”, **Table S2** for “miso Soup”, **Table S3** for “seaweed”, **Table S4** for “pickles”, **Table S5** for “green tea”, **Table S6** for “coffee”, **Table S7** for “mushrooms”.

The definitions of the five food groups other than those listed above are as following;

Fish

All foods items that corresponded to “PF_SEAFD_HI” and “PF_SEAFD_LOW” in the “variable_name” of Food Patterns Equivalent database (FPED) corresponded were assumed as foods corresponding to “fish” in the USDA Food and Nutrient Database for Dietary Studies (FNDDS).

Green and yellow vegetables

All foods items that corresponded to “V_DRKGR” and “V_REDOR_TOTAL” in the “variable_name” of FPED corresponded were assumed as foods corresponding to “green and yellow vegetables” in the FNDDS.

Soybeans and soy products

All foods items that corresponded to “PF_SOY” and “PF_LEGUMES” in the “variable_name” of FPED corresponded were assumed as foods corresponding to “green and yellow vegetables” in the FNDDS.

Beef and pork

All foods items that corresponded to “PF_MEAT” in the “variable_name” of FPED corresponded were assumed as foods corresponding to “beef and pork” in the FNDDS.

Fruits

All foods items that corresponded to “F_TOTAL” and “F_OTHER” in the “variable_name” of FPED corresponded were assumed as foods corresponding to “fruits” in the FNDDS.

Table S1 Foods corresponding to rice in the FNDDS

Food code	Main food description	Additional food description	WWEIA Category description
56205000	Rice, cooked, NFS		rice
56205060	Rice, cooked, with milk		rice
56205190	Rice, white, cooked, glutinous	sushi rice;sticky rice	rice
56205002	Rice, white, cooked, made with oil	basmati or jasmine rice	rice
56205008	Rice, white, cooked, no added fat	basmati or jasmine rice	rice
56205001	Rice, white, cooked, NS as to fat	basmati or jasmine rice	rice
56205006	Rice, white, cooked, made with margarine	made with shortening;basmati or jasmine rice	rice
56205004	Rice, white, cooked, made with butter	made with animal fat or meat drippings; basmati or jasmine rice	rice
56205014	Rice, brown, cooked, made with butter	made with animal fat or meat drippings	rice
56205016	Rice, brown, cooked, made with margarine	made with shortening	rice
56205018	Rice, brown, cooked, no added fat	made without fat	rice
56205011	Rice, brown, cooked, NS as to fat		rice
56205170	Yellow rice, cooked, fat added		rice
56205150	Yellow rice, cooked, no added fat		rice
56205130	Yellow rice, cooked, NS as to fat		rice
56205320	Rice, white and wild, cooked, fat added	Uncle Ben's Long Grain and Wild Rice flavored, all flavors;flavored white and wild rice, NFS	rice
56205300	Rice, white and wild, cooked, no added fat	Uncle Ben's Long Grain and Wild Rice flavored, all flavors	rice
56205330	Rice, white and wild, cooked, NS as to fat	Uncle Ben's Long Grain and Wild Rice flavored, all flavors	rice
56205340	Rice, brown and wild, cooked, fat added		rice

56205310	Rice, brown and wild, cooked, no added fat		rice
56205350	Rice, brown and wild, cooked, NS as to fat		rice
56205215	Rice, wild, 100%, cooked, fat added		rice
56205210	Rice, wild, 100%, cooked, no added fat		rice
56205205	Rice, wild, 100%, cooked, NS as to fat		rice
56205012	Rice, brown, cooked, fat added, made with oil		rice
56205410	Rice, white, cooked with fat, Puerto Rican style	Arroz blanco	rice
56205007	Rice, white, cooked, fat added, NS as to fat type	basmati or jasmine rice	rice
56205017	Rice, brown, cooked, fat added, NS as to fat type		rice
56205101	Congee.	Conjee;Asian;rice porridge or gruel	rice
56205070	Rice, sweet, cooked with honey		rice

Table S2 Foods corresponding to miso soup in the FNDDS

Food code	Main food description	Additional food description	WWEIA Category description
41601070	Soybean soup, miso broth	tofu soup; bean curd soup	Soups

Table S3 Foods corresponding to seaweed in the FNDDS

Food code	Main food description	Additional food description	WWEIA Category description
75647000	Seaweed soup	sea moss;laver;kelp	Soups
75232000	Seaweed, dried	sea moss, kelp;laver	Not included in a food category
75513010	Seaweed, pickled	sea moss;laver;kelp	Olives, pickles, pickled vegetables
75127500	Seaweed, raw	kelp;laver;sea moss;blanched	Other vegetables and combinations
75232120	Seaweed, cooked, fat added	NS as to fat added;from restaurant	Other vegetables and combinations
75232110	Seaweed, cooked, no added fat	cooking spray	Other vegetables and combinations

Table S4 Foods corresponding to pickles in the FNDDS

Food code	Main food description	Additional food description	WWEIA Category description
75511100	Pickles, NFS	mixed pickles	Olives, pickles, pickled vegetables
75500210	Beets, pickled	beet salad; pickled beets with onions	Olives, pickles, pickled vegetables
75502010	Cauliflower, pickled		Olives, pickles, pickled vegetables
75500510	Celery, pickled		Olives, pickles, pickled vegetables
75503080	Eggplant, pickled		Olives, pickles, pickled vegetables
75505000	Mushrooms, pickled		Olives, pickles, pickled vegetables
75507000	Okra, pickled		Olives, pickles, pickled vegetables
75511020	Peppers, pickled	red, green, and yellow	Olives, pickles, pickled vegetables
75503010	Pickles, dill	Sour	Olives, pickles, pickled vegetables
75503040	Pickles, sweet	bread and butter;gherkin	Olives, pickles, pickled vegetables
75503020	Relish, pickle	mustard;relish, NFS;hot dog;sweet	Olives, pickles, pickled vegetables
75534030	Turnip, pickled		Olives, pickles, pickled vegetables
75515100	Vegetables, pickled	Giardiniera	Olives, pickles, pickled vegetables
75535000	Zucchini, pickled		Olives, pickles, pickled vegetables
75511040	Pepper, hot, pickled	hot pepper, NFS;pickled jalapeno pepper	Olives, pickles, pickled vegetables
75502510	Cabbage, red, pickled	sweet and sour red cabbage	Olives, pickles, pickled vegetables
75503085	Ginger root, pickled	gari;sushi ginger	Olives, pickles, pickled vegetables
75500110	Green beans, pickled		Olives, pickles, pickled vegetables
74205020	Tomato, green, pickled		Olives, pickles, pickled vegetables
75512010	Radishes, pickled, Hawaiian style		Olives, pickles, pickled vegetables
75502500	Cabbage, fresh, pickled, Japanese style		Olives, pickles, pickled vegetables
75502520	Kimchi	Kim Chee	Olives, pickles, pickled vegetables
75510050	Olive tapenade	olive spread	Olives, pickles, pickled vegetables
75510020	Olives, black	purple;kalamata	Olives, pickles, pickled vegetables
75510010	Olives, green		Olives, pickles, pickled vegetables

75510000	Olives, NFS		Olives, pickles, pickled vegetables
75510030	Olives, stuffed		Olives, pickles, pickled vegetables
75501010	Relish, corn	vegetable relish	Olives, pickles, pickled vegetables
75230000	Sauerkraut		Olives, pickles, pickled vegetables

Table S5 Foods corresponding to green tea in the FNDDS

Food code	Main food description	Additional food description	WWEIA Category description
92303010	Tea, hot, leaf, green	tea bags;unsweetened;brewed;green tea blends;Japanese green tea	Tea
92303100	Tea, hot, leaf, green, decaffeinated	unsweetened;Japanese green tea;rice tea;tea bags;brewed;white tea;green tea blends	Tea
92305900	Tea, iced, instant, green, unsweetened	liquid concentrate;green and fruit, herbal, or white tea blends;powdered mix;decaffeinated;white tea;herbal tea	Tea
92305910	Tea, iced, instant, green, pre-sweetened with sugar	white tea;decaffeinated;liquid concentrate;powdered mix;green and fruit, herbal, or white tea blends;herbal tea;NS as to sweetener	Tea
92305920	Tea, iced, instant, green, pre-sweetened with low calorie sweetener	liquid concentrate;powdered mix;herbal tea;light, reduced calorie, diet, sugar free;decaffeinated;green and fruit, herbal, or white tea blends white tea	Tea
92308500	Tea, iced, brewed, green, pre-sweetened with sugar	green and fruit, herbal, or white tea blends;white tea;NS as to regular or decaffeinated;NS as to sweetener;herbal tea;leaf, bag, or vending	Tea
92308510	Tea, iced, brewed, green, pre-sweetened with low calorie sweetener	green and fruit, herbal, or white tea blends;NS as to regular or decaffeinated;herbal tea;white tea;leaf, bag, or vending	Tea
92308520	Tea, iced, brewed, green, unsweetened	NS as to regular or decaffeinated;white tea;leaf, bag, or vending;green and fruit, herbal, or white tea blends;herbal tea	Tea
92308530	Tea, iced, brewed, green, decaffeinated, pre-sweetened with sugar	leaf, bag, or vending;green and fruit, herbal, or white tea bleads;NS as to sweetener;herbal tea;white tea	Tea
92308540	Tea, iced, brewed, green, decaffeinated, pre-sweetened with low calorie sweetener	green and fruit, herbal, or white tea blends;leaf, bag, or vending;white tea;herbal tea	Tea
92308550	Tea, iced, brewed, green, decaffeinated, unsweetened	white tea;green and fruit, herbal, or white tea blends;herbal tea;leaf, bag, or vending	Tea
92309500	Tea, iced, bottled, green	white tea;snapple;can, carton, or fountain;decaffeinated;green and fruit, herbal, or white tea blends;herbal tea	Tea

92309510	Tea, iced, bottled, green, diet	can, carton, or fountain;white tea;light, reduced calorie, or sugar free;Diet Snapple;herbal tea;green and fruit, herbal, or white tea blends;. decaffeinated	Tea
92309520	Tea, iced, bottled, green, unsweetened	can, carton, or fountain;decaffeinated;green and fruit, herbal, or white tea blends;herbal tea;white tea	Tea

Table S6 Foods corresponding to coffee in the FNDDS

Food code	Main food description	Additional food description	WWEIA Category description
92101000	Coffee, brewed	coffee singles, bags, pods, or K-cups; coffee, brewed, NS as to regular or decaffeinated	Coffee
92101600	Coffee, Turkish		Coffee
92101610	Coffee, espresso	demi-tasse	Coffee
92101630	Coffee, espresso, decaffeinated	demi-tasse	Coffee
92101700	Coffee, brewed, flavored	coffee singles, bags, pods, or K-cups	Coffee
92101800	Coffee, Cuban		Coffee
92101810	Coffee, macchiato		Coffee
92101900	Coffee, Latte	NS as to regular or decaffeinated;plain or unflavored;2% or whole milk	Coffee
92102400	Iced Coffee, brewed	unsweetened;NS as to regular or decaffeinated;coffee singles, bags, pods, or K-cups	Coffee
92102500	Coffee, Iced Latte	NS as to regular or decaffeinated;plain or unflavored;2% or whole milk	Coffee
92103000	Coffee, instant, reconstituted	NS as to regular or decaffeinated;made from liquid concentrate;powdered mix	Coffee
92111010	Coffee, brewed, decaffeinated	coffee singles, bags, pods, or K-cups	Coffee
92152000	Coffee and chicory, brewed		Coffee
92161001	Coffee, Cappuccino, nonfat	low fat, fat free, skim or 1% milk; NS as to regular or decaffeinated	Coffee
92162000	Coffee, Cappuccino, decaffeinated	2% or whole milk	Coffee
92171000	Coffee, bottled/canned	all brands;from carton;all varieties;NS as to brand or variety;all flavors	Coffee
92201010	Coffee substitute		Coffee
92101910	Coffee, Latte, decaffeinated	2% or whole milk; plain or unflavored	Coffee
92101904	Coffee, Latte, flavored	2% or whole milk;flavors other than chocolate;NS as to regular or decaffeinated	Coffee
92101901	Coffee, Latte, nonfat	NS as to regular or decaffeinated;plain or unflavored;low fat, fat free, skim or 1% milk;sugar free	Coffee
92101820	Coffee, macchiato, sweetened	caramel macchiato	Coffee
92100000	Coffee, NS as to type		Coffee
92130010	Coffee, pre-lightened	NS as to regular or decaffeinated;from vending machine;unsweetened or sugar-free	Coffee
92100500	Coffee, NS as to brewed or instant	coffee singles, bags, pods, or K-cups	Coffee
92102401	Iced Coffee, brewed, decaffeinated	unsweetened; coffee singles, bags, pods, or K-cups	Coffee
92101850	Coffee, cafe con leche	beverage made with equal amounts of coffee and milk, sugar added	Coffee

92152010	Coffee and chicory, brewed, decaffeinated		Coffee
92171010	Coffee, bottled/canned, light	all flavors;NS as to brand or variety;from carton;all brands;all varieties	Coffee
92101965	Coffee, Cafe Mocha, decaffeinated	mocha latte;chocolate flavored;2% or whole milk	Coffee
92101955	Coffee, Cafe Mocha, nonfat	low fat, fat, free, skim or 1% milk;mocha latte;NS as to regular or decaffeinated;chocolate flavored	Coffee
92162001	Coffee, Cappuccino, decaffeinated, nonfat	low fat, fat free, skim or 1% milk	Coffee
92130011	Coffee, decaffeinated, pre-lightened	from vending machine;unsweetened or sugar-free	Coffee
92102510	Coffee, Iced Latte, decaffeinated	2% or whole milk; plain or unflavored	Coffee
92102503	Coffee, Iced Latte, flavored	NS as to regular or decaffeinated;2% or whole milk;flavors other than chocolate	Coffee
92102501	Coffee, Iced Latte, nonfat	NS as to regular or decaffeinated;sugar free;low fat, fat free, skim or 1% milk;plain or unflavored	Coffee
92114000	Coffee, instant, decaffeinated, reconstituted	powdered mix	Coffee
92101917	Coffee, Latte, decaffeinated, flavored	flavors other than chocolate;2% or whole milk	Coffee
92101911	Coffee, Latte, decaffeinated, nonfat	sugar free;plain or unflavored;low fat, fat free, skim or 1% milk	Coffee
92101905	Coffee, Latte, nonfat, flavored	flavors other than chocolate;low fat, fat free, skim or 1% milk;NS as to regular or decaffeinated	Coffee
92130020	Coffee, pre-sweetened with sugar	NS as to type of sweetener;from vending machine;NS as to regular or decaffeinated	Coffee
92101930	Frozen coffee drink, decaffeinated	Starbuck's Frappuccino; 2% or whole milk; plain, unflavored, or flavors other than chocolate	Coffee
92102000	Frozen mocha coffee drink	McDonald's McCafe Frappe Mocha;Starbuck's Mocha Frappuccino;chocolate or cocoa flavored;2% or whole milk;NS as to regular or decaffeinated	Coffee
92111000	Coffee, NS as to brewed or instant, decaffeinated	coffee singles, bags, pods, or K-cups	Coffee
92101851	Coffee, cafe con leche, decaffeinated	beverage made with equal amounts of decaffeinated coffee and milk, sugar added	Coffee
92101500	Coffee, brewed, blend of regular and decaffeinated	coffee singles, bags, pods or K-cups;half-caf;reduced caffeine	Coffee
92101970	Coffee, Cafe Mocha, decaffeinated, nonfat	low fat, fat free, skim or 1% milk;chocolate flavored;mocha latte	Coffee
92161002	Coffee, Cappuccino, with non-dairy milk	NS as to regular or decaffeinated;almond, coconut, rice, or soy milk	Coffee
92130021	Coffee, decaffeinated, pre-sweetened with sugar	NS as to type of sweetener;from vending machine	Coffee

92102610	Coffee, Iced Cafe Mocha, decaffeinated	2% or whole milk;iced mocha latte;chocolate flavored	Coffee
92102601	Coffee, Iced Cafe Mocha, nonfat	NS as to regular or decaffeinated;iced mocha latte;chocolate flavored;low fat, fat free, skim or 1% milk	Coffee
92102513	Coffee, Iced Latte, decaffeinated, flavored	2% or whole milk; flavors other than chocolate	Coffee
92102511	Coffee, Iced Latte, decaffeinated, nonfat	sugar free;low fat, fat free, skim or 1% milk;plain or unflavored	Coffee
92102504	Coffee, Iced Latte, nonfat, flavored	flavors other than chocolate;low fat, fat free, skim or 1% milk;NS as to regular or decaffeinated	Coffee
92101918	Coffee, Latte, decaffeinated, nonfat, flavored	flavors other than chocolate;low fat, fat free, skim or 1% milk	Coffee
92101903	Coffee, Latte, with non-dairy milk	almond, coconut, rice, or soy milk;NS as to regular or decaffeinated;plain or unflavored	Coffee
92101931	Frozen coffee drink, decaffeinated, nonfat	low fat, fat free, skim or 1% milk;sugar free;Starbuck's Skinny Frappuccino;plain, unflavored, or flavors other than chocolate	Coffee
92102060	Frozen mocha coffee drink, decaffeinated	chocolate or cocoa flavored;2% or whole milk;Starbuck's Mocha Frappuccino	Coffee
92102450	Iced Coffee, pre-lightened and pre-sweetened	NS as to regular or decaffeinated;McDonald's Iced Coffee;from vending;beverage dispensers	Coffee
92101960	Coffee, Cafe Mocha, with non-dairy milk	mocha latte;chocolate flavored;NS as to regular or decaffeinated	Coffee
92102611	Coffee, Iced Cafe Mocha, decaffeinated, nonfat	iced mocha latte; low fat, fat free, skim or 1% milk; chocolate flavored	Coffee
92102514	Coffee, Iced Latte, decaffeinated, nonfat, flavored	low fat, fat free, skim or 1% milk; flavors other than chocolate	Coffee
92102502	Coffee, Iced Latte, with non-dairy milk	NS as to regular or decaffeinated;plain or unflavored;almond, coconut, rice, or soy milk	Coffee
92104000	Coffee, instant, 50% less caffeine, reconstituted	blend of regular and decaf;powdered mix;half-caf;reduced caffeine	Coffee
92121010	Coffee, instant, pre-sweetened with sugar, reconstituted	powdered mix; NS as to sweetener	Coffee
92101913	Coffee, Latte, decaffeinated, with non-dairy milk	almond, coconut, rice, or soy milk; plain or unflavored	Coffee
92101906	Coffee, Latte, with non-dairy milk, flavored	almond, coconut, rice, or soy milk; flavors other than chocolate; NS as to regular or decaffeinated	Coffee
92130000	Coffee, pre-lightened and pre-sweetened with sugar	From vending machine; NS as to regular or decaffeinated; NS as to type of sweetener	Coffee
92130030	Coffee, pre-sweetened with low calorie sweetener	NS as to regular or decaffeinated;from vending machine	Coffee
92101975	Coffee, Cafe Mocha, decaffeinated, with non-dairy milk	mocha latte;chocolate flavored;almond, coconut, rice, or soy milk	Coffee

92130001	Coffee, decaffeinated, pre-lightened and pre-sweetened with sugar	from vending machine;NS as to type of sweetener	Coffee
92102602	Coffee, Iced Cafe Mocha, with non-dairy milk	almond, coconut, rice, or soy milk;mocha latte;NS as to regular or decaffeinated;chocolate flavored	Coffee
92102512	Coffee, Iced Latte, decaffeinated, with non-dairy milk	almond, coconut, rice, or soy milk; plain or unflavored	Coffee
92102505	Coffee, Iced Latte, with non-dairy milk, flavored	Flavors other than chocolate;almond, coconut, rice, or soy milk;NS as to regular or decaffeinated	Coffee
92101919	Coffee, Latte, decaffeinated, with non-dairy milk, flavored	almond, coconut, rice, or soy milk; flavors other than chocolate	Coffee
92102612	Coffee, Iced Cafe Mocha, decaffeinated, with non-dairy milk	almond, coconut, rice, or soy milk;chocolate flavored;mocha latte	Coffee
92102515	Coffee, Iced Latte, decaffeinated, with non-dairy milk, flavored	almond, coconut, rice, or soy milk; flavors other than chocolate	Coffee
92121000	Coffee, instant, pre-lightened and pre-sweetened with sugar, reconstituted	Maxwell House International, flavors other than chocolate, cocoa, or mocha;NS as to sweetener;powdered mix	Coffee
92130005	Coffee, pre-lightened and pre-sweetened with low calorie sweetener	NS as to regular or decaffeinated;from vending machine	Coffee
92102100	Frozen mocha coffee drink, decaffeinated, nonfat, with whipped cream	Starbuck's Skinny Mocha Frappuccino;chocolate or cocoa flavored;low fat, fat free, skim or 1% milk;NS as to with or without whipped cream;sugar free	Coffee
92121020	Coffee, mocha, instant, pre-lightened and pre-sweetened with sugar, reconstituted	NS as to sweetener;powdered mix;Maxwell House International Coffee flavors, Chocolate, Cocoa, or Mocha;coffee and cocoa mix	Coffee
92130006	Coffee, decaffeinated, pre-lightened and pre-sweetened with low calorie sweetener	from vending machine	Coffee
92121001	Coffee, instant, decaffeinated, pre-lightened and pre-sweetened with sugar, reconstituted	Maxwell House International, flavors other than chocolate, cocoa, or mocha; powdered mix	Coffee
92101938	Frozen coffee drink, decaffeinated, with non-dairy milk and whipped cream	NS as to with or without whipped cream;plain, unflavored, or flavors other than chocolate;almond, coconut, rice, or soy milk	Coffee
92102050	Frozen mocha coffee drink, with non-dairy milk and whipped cream	NS as to regular or decaffeinated;almond, coconut, rice, or soy milk;chocolate or cocoa flavored;NS as to with or without whipped cream	Coffee
92121040	Coffee, instant, pre-lightened and pre-sweetened with low calorie sweetener,	flavors other than chocolate, cocoa, or mocha;Maxwell House International Sugar Free Coffee;powdered mix;sugar free	Coffee

	reconstituted		
92121041	Coffee, instant, decaffeinated, pre-lightened and pre-sweetened with low calorie sweetener, reconstituted	sugar free;powdered mix;Maxwell House International Sugar Free Coffee;flavors other than chocolate, cocoa, or mocha	Coffee
92203000	Cereal beverage	Pero	Coffee
92203110	Cereal beverage with beet roots, from powdered instant	Cafix	Coffee
92202010	Chicory beverage		Coffee
92101920	Frozen coffee drink	Plain, unflavored, or flavors other than chocolate;2% or whole milk;McDonald's McCafe Frappe;Starbuck's Frappuccino;NS as to regular or Decaffeinated;Dunkin Donut's Coffee Coolatta	Coffee
92101921	Frozen coffee drink, nonfat	plain, unflavored, or flavors other than chocolate;NS as to regular or decaffeinated;low fat, fat free, skim or 1% milk;sugar free;Dunkin Donut's Coffee Coolatta;Starbuck's Skinny Frappuccino	Coffee
92101923	Frozen coffee drink, with non-dairy milk	almond, coconut, rice, or soy milk;NS as to regular or decaffeinated;plain, unflavored or flavors other than chocolate	Coffee
92101925	Frozen coffee drink, with whipped cream	2% or whole milk;Starbuck's Coffee Frappuccino;plain, unflavored, or flavors other than chocolate;NS as to with or without whipped cream;McDonald's McCafe Frappe;NS as to regular or decaffeinated	Coffee
92101933	Frozen coffee drink, decaffeinated, with non-dairy milk	plain, unflavored, or flavors other than chocolate;almond, coconut, rice, or soy milk	Coffee
92101935	Frozen coffee drink, decaffeinated, with whipped cream	NS as to with or without whipped cream;2% or whole milk;Starbuck's Coffee Frappuccino;plain, unflavored, or flavors other than chocolate	Coffee
92101936	Frozen coffee drink, decaffeinated, nonfat, with whipped cream	plain, unflavored, or flavors other than chocolate;Starbuck's Skinny Frappuccino;low fat, fat free, skim or 1% milk;sugar free;NS as to with or without whipped cream	Coffee
92101950	Coffee, Cafe Mocha	chocolate flavored;2% or whole milk;NS as to regular or decaffeinated;mocha latte	Coffee
92102020	Frozen mocha coffee drink, with non-dairy milk	almond, coconut, rice, or soy milk;NS as to regular or decaffeinated;chocolate or cocoa flavored	Coffee
92102030	Frozen mocha coffee drink, with whipped cream	McDonald's McCafe Frappe Mocha;NS as to with or without whipped cream;Starbuck's Mocha Frappuccino;chocolate or cocoa flavored;NS as to regular or decaffeinated;2% or whole milk	Coffee

92102040	Frozen mocha coffee drink, nonfat, with whipped cream	chocolate or cocoa flavored;low fat, fat free, skim or 1% milk;NS as to regular or decaffeinated;NS as to with or without whipped cream;Starbuck's Skinny Mocha Frappuccino;sugar free	Coffee
92102070	Frozen mocha coffee drink, decaffeinated, nonfat	sugar free;Starbuck's Skinny Mocha Frappuccino;low fat, fat free, skim, or 1% milk;chocolate or cocoa flavored	Coffee
92102090	Frozen mocha coffee drink, decaffeinated, with whipped cream	2% or whole milk;NS as to with or without whipped cream;Starbuck's Mocha Frappuccino;chocolate or cocoa flavored	Coffee
92102110	Frozen mocha coffee drink, decaffeinated, with non-dairy milk and whipped cream	chocolate or cocoa flavored;NS as to with or without whipped cream;almond, coconut, rice, or soy milk	Coffee

Table S7 Foods corresponding to mushrooms in the FNDDS

Food code	Main food description	Additional food description	WWEIA Category description
75414030	Fried mushrooms	battered or breaded	Fried vegetables
99997515	Mushrooms, cooked, as ingredient		Not included in a food category
75115000	Mushrooms, raw		Other vegetables and combinations
75219011	Mushrooms, fresh, cooked, no added fat	cooking spray	Other vegetables and combinations
75219020	Mushrooms, NS as to form, cooked	with or without fat	Other vegetables and combinations
75219021	Mushrooms, fresh, cooked, fat added, NS as to fat type	NS as to fat added	Other vegetables and combinations
75219023	Mushrooms, canned, cooked	with or without fat; frozen	Other vegetables and combinations
75219033	Mushrooms, fresh, cooked with oil		Other vegetables and combinations
75219034	Mushrooms, fresh, cooked with butter or margarine	animal fat; shortening; from restaurant	Other vegetables and combinations
75219100	Mushroom, Asian, cooked, from dried	shiitake	Other vegetables and combinations
75414020	Mushrooms, stuffed		Vegetable dishes

Table S8 Distribution of the Japanese Diet Index points

JDI[1)]	n	%
0	233	7.4%
1	860	27.4%
2	1086	34.6%
3	671	21.4%
4	224	7.1%
5	53	1.7%
6	11	0.4%
7	0	0.0%
8	0	0.0%
9	0	0.0%
Total	3138	100.0%

1) Japanese Diet Index

Table S9 Distribution of the Modified Japanese Diet Index points

mJDI[1)]	N	%
0	93	3.0%
1	439	14.0%
2	848	27.0%
3	861	27.4%
4	534	17.0%
5	246	7.8%
6	94	3.0%
7	18	0.6%
8	4	0.1%
9	1	0.0%
10	0	0.0%
11	0	0.0%
12	0	0.0%
Total	3138	100.0%

1) Modified Japanese Diet Index

Table S10 Distribution of the Weighted Japanese Diet Index points

wJDI[1)]	n	%
0	280	8.9%
1	351	11.2%
2	117	3.7%
3	627	20.0%
4	505	16.1%
5	194	6.2%
6	486	15.5%
7	294	9.4%
8	134	4.3%
9	150	4.8%
Total	3138	100.0%

1) Weighted Japanese Diet Index
